# Effects of Minocycline on Neurological Outcomes In Patients With Acute Traumatic Brain Injury: A Pilot Study

**DOI:** 10.22037/ijpr.2019.1100677

**Published:** 2019

**Authors:** Neda Koulaeinejad, Kaveh Haddadi, saeid ehteshami, misagh shafizad, Ebrahim Salehifar, Omid Emadian, Reza Ali Mohammadpour, Shahram Ala

**Affiliations:** a *Department of Clinical Pharmacy, Faculty of Pharmacy, Mazandaran University of Medical Sciences, Sari, Iran.*; b *Department of Neurosurgery, Faculty of Medicine, Mazandaran University of Medical Sciences, Sari, Iran.*; c *Department of Pathology, Faculty of Medicine, Mazandaran University of Medical Sciences, Sari, Iran.*; d *Department of Biostatistics, Faculty of Health Sciences, Mazandaran University of Medical Sciences, Sari, Iran.*

**Keywords:** Neuron-specific enolase (NSE), S100B, Glasgow coma scale (GCS), Glasgow Outcome Scale-Extended (GOS-E), Minocycline, Acute traumatic brain injury

## Abstract

Traumatic brain injury (TBI) is a public health problem worldwide. Secondary damage of brain injury begins within a few minutes after the trauma and can last a long time. It can be reversible, unlike primary injury. Therefore, therapeutic intervention can be used. The aims of this study were to assess the effects of minocycline on neurological function and serum S100B protein and neuron-specific enolase (NSE) levels in patients with moderate to severe TBI. Patients with acute onset of TBI and surgical evacuation of hematoma were randomized to receive either minocycline 100 mg orally twice daily or placebo for 7 days. The primary outcomes included changes in level of S100B and NSE at different time points during the trial. Additionally, changes in Glasgow coma scale (GCS) score were evaluated. The Glasgow Outcome Scale-Extended (GOS-E) score at 6 months after injury was assessed in discharge patients. Thirty four patients were randomized into the placebo (n = 20) and treatment (n = 14) groups. There was a marginal statistically significant differences in the normalized value of S100B between groups (*p* < 0.1). The reduction in serum NSE level from baseline to day 5 was statistically significant (*p* = 0.01) in minocycline group while it was not significantly decrease in placebo group (*p* = 0.2). Also, GCS improvement over time within the minocycline group was significant (*p* = 0.04) while was not significant in placebo group (*p* = 0.11). The GOS-E scores were not significantly different between minocycline and placebo group. Based on this study, it seems that the use of minocycline may be effective in acute TBI.

## Introduction

Traumatic brain injury (TBI) is a public health problem in both industrialized and developing countries (1-3). TBI affects around 10 million people annually, leading to hospitalization or death worldwide (2). According to World Health Organization (WHO), TBI will be ahead of many diseases as the major cause of death and disability by the year 2020 (1). Neurocognitive dysfunction with lasting changes in cognition, motor function and personality occur in many survivors (4) and many patients cannot return to work post injury (3). Hence, TBI impose large direct and indirect cost to society.

Pathophysiological changes of TBI consist of two phases. The primary injury is caused by the mechanical damage from shearing, tearing, and/or stretching of neurons, axons, glia, and blood vessels. This injury occurs at the exact moment of insult and can be diffuse, focal, or a combination of both with directs neural cell loss and necrotic cell death. In response to initial injury, the secondary injury occurs. This injury consists of excitotoxicity, oxidative stress, mitochondrial dysfunction, blood–brain barrier (BBB) disruption, and inflammation. These mechanisms cause to worsening the progressive outcome of TBI. Secondary damage begins within a few minutes after the trauma and can last for days, months, or years. It is believed that secondary injury can be reversible, unlike primary injury. Therefore, therapeutic intervention can be used (5-9). 

Minocycline is a tetracycline derivative antibiotic. In addition, it has been recognized for its neuroprotective properties in the experimental and clinical studies of neurodegenerative diseases (10, 11) such as TBI (12-15). Minocycline has a long half-life (16–18 h) and easily penetrate to the BBB because of its highly lipophilic molecule (16). Minocycline has broad anti-inflammatory properties through reducing the secretion of proinflammatory cytokines, chemokines, lipid mediators of inflammation, matrix metalloproteases (MMPs) and nitric oxide (NO) by modulating microglia cells (10). Other effects of minocycline include reducing perihemorrhagic edema, BBB dysfunction, iron-mediated oxidative stress , and apoptosis (10, 17). 

In the last two decades, to predict the severity or the potential outcome of head injuries, different neuromarkers, such as neuron-specific enolase (NSE) and S100B, have been studied (18-23). S100B is a calcium-binding protein, can be found predominantly in glial cells with a half-life of 30 min (19, 20, 24). NSE is a glycolytic enzyme family predominantly located in neurons and neuroectodermal cells with a half-life of approximately 24 hours (18). After TBI the level of these neurobiochemical markers increased in serum and correlated with outcome (23, 25). Given these points, we used both S100B, which is a marker of astroglial tissue, and NSE, which is a marker of neuronal tissue, for our study to provide a complete spectrum of neuroglial injury after TBI.

The aims of this study was to examine the short term effects of minocycline on neurological function and serum S100B and NSE levels in patients with TBI.

*Methods*

This study was registered at the Iranian registry of clinical trials (IRCT) with registration number IRCT201602063014N12 (The full trial protocol could be accessed online at www.irct.ir). 

Also, all the procedures in this study was approved by the medical ethics committee at Mazandaran University of Medical Sciences (ethical approval number: IR.MAZUMS.REC.95-2327) and carried out according to the declaration of Helsinki and subsequent revisions.

Because almost all participants were unconscious at the time of study entry, the informed consent was obtained from their legally authorized representative who signed the surgical consent form. If this person was not available other first-degree relatives of patient or patient’s spouse was filled the informed consent form. They were informed that they could leave the trial at any time. 

*Trial Design and Setting*

We performed a prospective randomized; double-blind pilot clinical trial in patients with moderate to severe TBI which was randomized into placebo and active treatment group by simple randomization procedure using a table of random numbers. This study was undertaken between August 2016 and November 2017 at Imam Khomeini General Hospital and educational center of Mazandaran University of Medical Sciences, Sari, Iran. This hospital is a tertiary-care multispecialty referral medical center with an active trauma and neurosurgical services, covering all patients in Mazandaran province in northern Iran. To prepare a placebo capsule, we ordered the same capsule shell as the minocycline capsule to Iran Gelatin Capsule Co. and filled it by glucose powder. The study drugs were placed in sealed, opaque, and similar drug containers and also administered to the participants. The randomization was performed by assigning the random numbers from random number tables to the treatment conditions. This procedure was carried out by a clinical pharmacist who was not involved in the subsequent trial procedure. The patients, healthcare providers and investigators were all blinded to the randomization. One investigator assessed inclusion and exclusion criteria and provided the minocycline or placebo to the nursing staff. Unmasking occurred after termination of the trial procedures and the person who analyzes the study data was not blinded.

*Patients*

Patients who met the following criteria were included in the study: Hospital admission in the first 24 h of injury, 18 to 90 years of age and both sexes with moderate to severe traumatic brain injury (GCS score ≤ 12) who has surgical evacuation of hematoma within 12 h after admission.

The exclusion criteria were hypersensitivity to tetracycline or minocycline medicine, Pregnant and breast feeding women, history of systemic lupus erythematosus (SLE), history of receiving chronic steroid treatment and isotretinoin, Pre-existing hepatic (AST, ALT greater than 3 times the upper limit of normal) or renal failure (BUN/ Creatinine 20:1; creatinine > 2 mg/dL), Significant leucopoenia (white blood cell count less than 0.5 times the lower limit of normal), Thrombocytopenia (platelets < 75,000/mm^3^), incidence of pseudomembranous colitis and Patients who expired 48 hours after injury.

Patients who were admitted to the emergency room were managed according to an institutional protocol based on the Brain Trauma Foundation Guidelines (26) such as adequate oxygenation, BP support, vital sign, and temperature monitoring. Emergency surgical treatment was based upon neurologic status and findings on head computed tomography (CT) criteria. Once patients were stable and inclusion criteria were met, they were randomized to receive either oral capsules of minocycline (Ranbaxy Pharmaceuticals Inc. USA) or placebo in addition to the standard treatment. 

The minocycline dose was 100 mg, twice daily, either orally or through a nasogastric tube for 7 days. 

The first dose was begun within 24 hof admission. The powder of capsule was dispersed and dissolved in a plate by approximately 5 mL of water and the contents were drawn up to the syringe and immediately administered via nasogastric tube. The plate and syringe were rinsed with a plenty of water and the flush contents were also emptied into nasogastric tube.

*Outcomes and data collection*

The characteristics (Age, Sex, mechanism of injury, severity of TBI) and vital signs of patients were assessed at baseline. Laboratory values and physiological variables (blood pressure, heart rate, respiratory rate, temperature, creatinine, and electrolytes) were also documented during the study period. The Acute Physiologic and Chronic Health Evaluation (APACHE II) score were assessed in all patients at baseline (27). Patients were evaluated for the severity of trauma by injury severity scoring (ISS) at the beginning of the study. The range of ISS is 0 to 75 point based on worst injury of six body systems (28). Additionally, the severity of brain injury was assessed using the Rotterdam CT score based on the admission of CT scan by a neurosurgeon that was blinded to the treatment assignment. It includes degree of basal cistern compression, midline shift, epidural hematomas, and intraventricular and/or subarachnoid blood. Completely normal appearing scan has a Rotterdam score of 1 and the worse possible score is 6 (29). 

The primary outcomes were the changes in levels of S100B and NSE. In order to measure these neurobiochemical markers, the blood samples were collected from day 1 to day 5. The samples were centrifuged in the central hospital laboratory and the serums were extracted and placed in the freezer at -70 °C until analysis. S100B was assessed on days 1 (before intervention), 2, 3, and 4 (after intervention) while NSE was assessed on day 1 (before intervention) and 5 (after intervention). These variables were measured by a commercially available enzyme-linked immunoabsorbent assay (ELISA) kit (DiaMetra, Milano, Italy), according to the manufacturer′s instructions (30). In addition, the changes in Glasgow Coma Scale (GCS) score were evaluated during 5 days study. GCS values were determined and recorded by neurosurgery service on morning rounds. The secondary outcomes were Length of hospital and ICU stay. Also, we used the Glasgow Outcome Scale-Extended (GOS-E) score for evaluating the recovery level of patients (31). One investigator interviewed with family member of the discharged patients at 6 months after the injury. 

*Statistical analysis*

All data analysis was performed using Statistical Package for Social Sciences (SPSS) software version 21 (SPSS Inc., Chicago, IL). For qualitative and quantitative variables, we used Frequency (percent) and mean (Standard Devi­ation), respectively. In addition, we presented non-normal­ variables with median (IQR: inter-quartile range). 

The normality of variables was tested by the Kolmogorov-Smirnov one-sample test. Comparisons between the two study groups at baseline were performed using chi-square or Fisher^,^s exact test for categorical data and the Independent samples t-test or Mann-Whitney U test for continuous data. General linear models (GLM) of normalized values of S100B between two groups were compared by repeated measurement ANOVA test. Time of evaluation was considered as the within-subject factor, and type of intervention (minocyclne or placebo) as the between-subject factor. The group time (interaction term) was considered as group differences (between minocycline and placebo groups) in their response over time. We tested Mauchley’s sphericity test for compound symmetry assumption. Wilcoxon signed rank test and Friedman^,^s test were used for analysis of NSE and GCS data, respectively. A *p* value of 0.05 or less was considered statistically significant, and a *p* value of less than 0.1 was considered marginally statistically significant.

## Results

*Study Participants*

**Figure 1:**
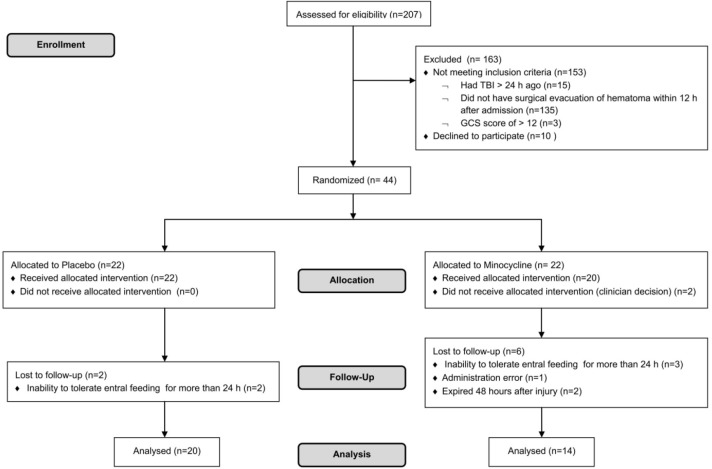
Diagram of participants (according to the guidelines of CONSORT 2010)

Of 207 patients who were screened for eligibility, 163 patients were excluded and 44 were enrolled. From 44 patients (22 in each study arm) who were randomized in the two groups, 8 and 2 patients were lost during allocation and follow up in the case and control groups, respectively. Finally, the data analysis was performed on 34 patients, who completed the study (Figure 1). 

Baseline demographic and clinical characteristics of minocycline and placebo groups were similar (Table 1). Also, laboratory values and physiological variables did not differ significantly between the two groups at baseline and during the study period.

*Serum biomarkers levels*

The mean of normalized value of serum S100B levels at baseline were 3.50±0.98 and 4.26±1.18 in the minocycline and placebo groups, respectively which were not statistically significant (*p* = 0.24). As shown in Table 2, there were statistically significant differences in time effect (*p* < 0.01). 

There was a marginal statistically significant differences in the normalized value of S100B between groups (*p* < 0.1). There was no interaction effect in the intervention group by placebo group (*p* > 0.05). 

The value of serum NSE level were not statistically significant at baseline (*p* = 0.84).As shown in Table 3, the reduction in serum NSE level from baseline to day 5 was statistically significant (*p* = 0.01) in minocycline group while it was not significantly decreased in placebo group (*p* = 0.2).

*Changes in GCS*

**Figure 2:**
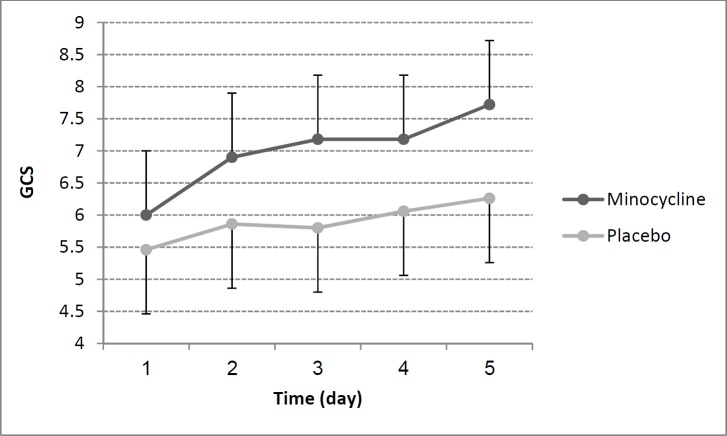
Glasgow Coma Scale (GCS) in minocycline and placebo groups

GCS values did not significantly differ between minocycline and placebo group at baseline (*p* = 0.52). As mentioned above we used Friedman’s test for compare GCS changes within the two study groups. GCS improvement over time within the minocycline group was significant (*p* = 0.04) while was not in placebo group (*p* = 0.11). GCS scores changes in minocycline and placebo group during 5 study days were shown in Figure 2. Additionally, there was a large correlation between reduction of NSE and the rise in GCS in all patients (r= -0.52, *p* = 0.006).

*Patient survival outcomes*

Of 34 patients, 23 cases were discharge and we performed GOS-E test at 6 months after the injury as secondary outcome. According to the results presented in Table 4, there were no statistically significant differences between minocycline and placebo group except for length of hospital stay where this difference was marginal statistically significant (*p* = 0.07). Hospital stay of patients in minocycline group was fewer days (14.35 ± 8.64) rather than placebo group (22.55 ±16.36).

## Discussion

Todays, TBI is a common devastating disease in young and the elderly people (32). Over the last decade several novel therapies used in animal models and human pilot studies for the treatment and prevention of secondary brain injury after TBI (33). The pathophysiological cascade following TBI included Glutamate release, intracellular calcium elevation, inflammatory response, apoptosis and etc. The investigators focus on these mechanisms because the treatment of the first insult injury is impossible (34). 

Minocycline is an FDA approved, second-generation tetracyclins antibiotic that demonstrates anti-inflammatory and neuroprotective effects in various experimental models of neurological disorders such as cerebral ischemia (35), Intracerebral hemorrhage (ICH) (36), amyotrophic lateral sclerosis (ALS) (37), Parkinson’s disease (38), Huntington′s disease (39), multiple sclerosis (MS) (40), Alzheimer’s disease (41), spinal cord injury (42), and TBI (12). 

**Table 1:** Demographic and clinical characteristics of patients at baseline

**Variable**	**All patients**	**Minocycline**	**Placebo**	***P*** **-value**
Number of patients	34	14	20	
Sex, n (% male)	30 (88.2)	12 (35.3)	18 (52.9)	0.70 *
Age, y (range)	42.50 ± 15.78 (18-73)	42.71 ± 16.22	42.35 ± 15.89	0.95 †
ISS (range)	20.70 ± 8.28 (4-42)	20.07 ± 7.86	21.15 ± 8.73	0.82 ‡
Mechanism of injury, n (%)				0.35 *
MVA	28 (82.35)	10 (71.42)	18 (90.00)	
FD	6 (17.64)	4 (28.57)	2 (10.00)	
GCS at hospital entry	7.64 ± 2.95	8.21 ± 2.93	7.25 ± 2.97	0.29 ‡
Severity of TBI, n (%)				0.20 *
moderate	11 (32.35)	6 (42.85)	5 (25.00)	
severe	23 (67.64)	8 (57.14)	15 (75.00)	
Rotterdam CT Score, n (%)				0.75 *
2	6 (17.64)	2 (14.28)	4 (20.00)	
3	6 (17.64)	2 (14.28)	4 (20.00)	
4	13 (38.23)	7 (50.00)	6 (30.00)	
5	9 (26.47)	3 (21.42)	6 (30.00)	
APACHE II (range)	18.38 ± 3.86 (8-29)	17.50 ± 3.71	19.00 ± 3.94	0.55 ‡
MAP (mmHg)	89.40 ± 11.69	88.26 ± 11.32	90.21 ± 12.16	0.64 †
T (°C)	37.47 ± 0.50	37.62 ± 0.50	37.35 ± 0.48	0.14 †
HR (beats/min)	95.72 ± 15.55	95.85 ± 16.97	95.63 ± 14.88	0.96 †
RR (breaths/min)	15.84 ± 2.62	15.50 ± 2.21	16.10 ± 2.92	0.52 †

Data are reported as mean ± SD or as number (percentages). *P *values were obtained by

ISS, Injury Severity Score; MVA, motor vehicle accident; FD, falling down; GCS, Glasgow Coma Scale; APACHE II, Acute Physiologic and Chronic Health Evaluation; MAP, Mean arterial pressure; T, Temperature; HR, Heart rate; RR, Respiratory rate.

* chi-square test or Fisher’s exact test as appropriate,

† Independent sampled t test,

‡ Mann-Whitney U test with significance set at *p *< .05.

**Table 2:** Normalized value of S100B on different days in both groups

**Variable**	**Group**	**Time**				**F Statistic**		
		**Day 1**	**Day 2**	**Day 3**	**Day 4**	**Time**	**Group**	**Time** * **Group**
Normalized value of S100B† (pg/ml)	Minocycline	3.50±0.98	2.80±1.50	2.65±1.77	2.46±2.23	13.11‡	2.91§	0.19
	Placebo	4.26±1.18	4.00±1.43	3.83±1.84	2.92±2.28			

* Interaction between time and group.

† Normalized value of S100B is Mean ± SD.

‡ Significance level less than 0.01.

§ Significance level less than 0.1.

**Table 3:** Original value of NSE on day 1 and 5 in both groups

**Variable**	**Group**	**Time**		***P*** ** value**
		**Day 1**	**Day 5**	
Original value of NSE* (ng/ml)	Minocycline	34.55 (20.93-42.14)	16.99 (12.96-35.37)	0.01†
	Placebo	39.83 (31.91-67.46)	24.42 (13.47-66.60)	0.20

* Original value of NSE is Median (IQR).

† Significance level less than 0.05.

**Table 4:** Survival outcomes in the two study groups

***P*** ** Value**	**Placebo**	**Minocycline**	**All**	**Secondary outcomes**
0.21 *	3.66 ± 1.82	5.00 ± 2.04	4.30 ± 2.00	GOS-E
0.07 †	22.55 ±16.36	14.35 ± 8.64	19.17 ± 14.15	Length of hospital stay, d
0.15 †	18.95 ± 16.35	11.78 ± 8.65	16.00 ± 14.01	Length of ICU stay, d
0.44 ‡	12/8	11/3	23/11	Discharge/Expired ratio

Results are reported as mean ± SD. *P* values were obtained by

* Mann-Whitney U test,

† Independent sampled t test,

‡ chi-square test with significance set at *p* < .05.

Because of the lacking data in human traumatic patients, the present study was performed to investigate whether adjunctive therapy with minocycline might have beneficial effect on clinical outcome and neuroinflammation after acute TBI. In the present study, we used the Rotterdam CT score and injury severity scoring to classify patients at baseline. Fortunately, the patients did not differ in this regard and it was the strength of our work.

The major finding of our study was the significantly greater reduction in NSE levels in the minocycline group compared with the placebo group. The original values of S100B were not normally distributed. So, the log transformation method was used to obtain the normalized values. We observed marginal statistically significant reduction in concentrations of S100B levels in minocycline group during three consecutive days (day 1, 2 and 3) after TBI. Although this reduction was not significant in term of statics but clinically it was important because positive results were seen in some patients. Also, the reduction in S100B levels was significant by the time, in both study groups. 

Our results were Consistent with previous findings. In 2012, Kovesdi et al. shown that Acute minocycline treatment in 4 consecutive days resulted a significant reduction in serum levels of all neuronal and damage markers like NSE, NF-H, Tau, S100B except for Glial fibrillary acidic protein (GFAP) in a rat model of mild blast traumatic brain injury. Similarly, minocycline treatment normalized the elevated tissue levels of these markers with the exception of GFAP (43). The ß subunit of S100 protein is more specific for the astroglial cells of the CNS. But it may be a serious source of error because it is also found in non nervous cells such as adipocytes, chondrocytes, and melanoma cells. On the other hand, it is affected by the age and gender of the patient. Additionally, the levels of S100B rise immediately after TBI, so it is a reliable marker of severity of primary injury. In contrast, the serum level of NSE is a maker of neuronal integrity and indicates secondary brain damage (20). 

The result of our study showed that the GOS-E scores did not significantly differ between two groups. Biomarkers are sensitive early measure of outcome compared with neurological outcome scales. So, because of inadequate numbers of patients, the use of NSE and S100B could be more powerful tools to detect outcome differences than GOS-E.

Our study provides further evidence for the neuroprotective effects of minocycline. Several animal studies have been shown that mincocycline is effective in TBI induced neuronal damage (12, 43-46). In models of acute TBI, minocycline reduced microglial activation (44, 45), perihematomal edema (14, 47), Neurological deficits, and brain atrophy (48). Currently, only two clinical trials have evaluated the effects of minocycline in TBI patients. Both of these studies investigated the effects of minocycline on chronic phase of TBI and carried out by Scott and colleagues. In 2016, they showed minocycline reduced microglial activation (49). But it is unclear whether the chronic microglial activation by TBI is harmful or beneficial. In 2018, Scott and colleagues suggested that microglial activation has a reparative effect in the chronic phase of TBI. They showed minocycline reduced microglial activation but increased axonal injury and neurodegeneration (50). 

However, minocycline treatment in clinical trials of other neurological conditions has shown varying results. Stroke trials showed improved neurological outcomes following minocycline in the acute phase (51-53). Also a clinical trial in spinal cord injury has shown positive results (54). But minocycline showed mixed and negative results in some neurodegenerative diseases trials (55-58). 

On the other hand, there is a Concern about the administration of minocycline in TBI. Minocycline could induce benign intracranial hypertension in several case reports. This rare adverse effect may manifest from two weeks to 18 months after commencing the medication (59). Although in this pilot study, our patients received minocycline therapy for 7 days, but this issue is important to consider in future larger clinical trials.

To the best of our knowledge, our study is the first randomized clinical trial which evaluated the effects of minocycline on Brain specific Serum Biomarkers in acute phase of TBI. Nevertheless, there are some limitations to our study. First, due to small sample size, the results of secondary outcomes are underpowered. However, the power of primary outcome, S100B results was acceptable and demonstrating efficacy for a larger trial. Second, we used the oral formulation of minocycline, just available in our country. Two patients did not receive allocated intervention because the surgical service did not allow administering the oral capsule. The oral availability of minocycline is under the influence of various factors but the injectable form of minocycline can produce more confident therapeutic serum concentration and it may be suitable for the studies on acute phases of TBI that the patients may be NPO. Finally, the basic form of GOS-E questionnaire was used in this study. The intermediate or advanced form, containing the compared functional outcomes of the patients at 3, 6, and 12 months, may be more accurate for long term assessment.

We conclude as a result of this pilot study that minocycline treatment was associated with improvement in neurological outcomes of acute TBI compared with placebo, warranting further clinical trials.
